# A phase 1 study evaluating the combination of an allosteric AKT inhibitor (MK-2206) and trastuzumab in patients with HER2-positive solid tumors

**DOI:** 10.1186/bcr3577

**Published:** 2013-11-19

**Authors:** Clifford Hudis, Charles Swanton, Yelena Y Janjigian, Ray Lee, Stephanie Sutherland, Robert Lehman, Sarat Chandarlapaty, Nicola Hamilton, Devika Gajria, James Knowles, Jigna Shah, Keith Shannon, Ernestina Tetteh, Daniel M Sullivan, Carolina Moreno, Li Yan, Hyo Sook Han

**Affiliations:** 1 Memorial Sloan-Kettering Cancer Center, 300 East 66th Street, New York, NY 10065, USA; 2 Royal Marsden Hospital, Fulham Road, London SW3 6JJ, UK; 3 Merck Sharp & Dohme Corp., 1 Merck Drive, Whitehouse Station, NJ 08889, USA; 4 H. Lee Moffitt Cancer Center and Research Institute, 12902 Magnolia Drive, Tampa, FL 33612, USA; 5 Breast Cancer Unit, St Albans City Hospital and Mount Vernon Breast Cancer Research Unit, Mount Vernon Cancer Centre, St Albans, Herts, UK

## Abstract

**Introduction:**

Trastuzumab is effective in human epidermal growth factor receptor 2 (HER2)-over-expressing breast and gastric cancers. However, patients may develop resistance through downstream signaling via the phosphatidylinositol 3-kinase (PI3K)/AKT pathway. This phase 1 trial was conducted to determine the safety and tolerability of the investigational AKT inhibitor MK-2206, to prepare for future studies to determine whether the combination with trastuzumab could inhibit compensatory signaling.

**Methods:**

Patients with HER2+ treatment-refractory breast and gastroesophageal cancer were enrolled. Treatment consisted of standard doses of intravenous trastuzumab and escalating dose levels of oral MK-2206 using either an every-other-day (45 mg and 60 mg QOD) or once-weekly (135 mg and 200 mg QW) schedule.

**Results:**

A total of 34 patients with HER2+ disease were enrolled; 31 received study-drug. The maximum tolerated dose (MTD) for MK-2206 in combination with trastuzumab was 60 mg for the QOD schedule and 135 mg for the QW schedule, although a true MTD was not established due to early termination of the trial. The most common treatment-emergent toxicities included fatigue, hyperglycemia, and dermatologic rash, consistent with prior experience; one death unrelated to treatment was reported. There was one complete response in a patient with metastatic breast cancer, one patient achieved a partial response, and 5 patients had stable disease for at least 4 months, despite progression on multiple prior trastuzumab- and lapatinib-based therapies. Results also indicate that trastuzumab does not affect the pharmacokinetics of MK-2206.

**Conclusions:**

Results suggest the AKT inhibitor MK-2206 can be safely combined with trastuzumab, and is associated with clinical activity, supporting further investigation.

**Trial registration:**

ClinicalTrials.gov; identifier: NCT00963547.

## Introduction

Approximately 20 to 25% of breast cancers [1,2] and 30% of gastric cancers [3] have overexpression and/or gene amplification of human epidermal growth factor receptor 2 (HER2), which serves as both a poor prognostic marker and a therapeutic target. HER2 amplification, detected by fluorescence *in situ* hybridization, or overexpression, determined by immunohistochemistry staining, predicts responsiveness to HER2-targeted agents, such as trastuzumab, lapatinib, and other newer agents. However, patients with metastatic HER2+ breast cancer or gastric cancer may have intrinsic resistance or develop partial or complete clinical resistance to HER2-targeted therapy during the course of treatment [4-6]. Understanding mechanisms of resistance could lead to the development of new strategies to overcome resistance in these patients. One mechanism of resistance to trastuzumab is mediated through activation of downstream signaling via the phosphatidylinositol-3 kinase (PI3K)/AKT pathway, which has been identified as a major determinant of trastuzumab resistance in breast cancer [7,8]. Several groups have shown that HER2+ breast cancer models that have been selected for trastuzumab resistance can be effectively targeted with PI3K or AKT inhibitors [9,10]. The potential to increase antitumor activity by blocking both AKT signaling and HER2 kinase has been further suggested by a study showing that combined inhibition of AKT and HER2 kinase activity is more effective than either one alone in HER2+ models [11].

MK-2206 is an investigational allosteric inhibitor of AKT that requires the PH domain of AKT for activity, but does not interact with the ATP binding pocket. As a result, MK-2206 is highly selective for AKT inhibition, has higher potency against recombinant human AKT1 and AKT2 isoforms than AKT3, has little off-target kinase activities, and is less vulnerable to feedback activation of AKT compared with ATP-competitive inhibitors [12]. In prior phase 1 studies, MK-2206 was tested in over 100 patients with solid tumors using an every other day (QOD) or once weekly (QW) dosing schedule [13]. Overall, MK-2206 was well tolerated at biologically active doses, with the maximum tolerable dose (MTD) established at 60 mg QOD; the MTD for the QW dosing schedule (expected to be less than 250 mg) was not established due to early discontinuation of the trial. The most significant dose-limiting toxicity (DLT) was rash, which was maculopapular in nature with a truncal distribution, and was distinct from the acneiform rash seen with epidermal growth factor receptor inhibitors. Pharmacokinetic testing revealed that MK-2206 has a long half-life (60 to 90 hours) and no substantial departure from dose proportionality, and preliminary evidence of clinical activity was seen in various tumors. Based on the preclinical rationale for the combination of MK-2206 and trastuzumab, as well as promising preclinical results, we conducted a phase 1 trial to evaluate the QOD and QW dosing schedules from earlier trials and to determine the MTD and recommended phase 2 dose for MK-2206, administered in combination with standard doses of trastuzumab. We also assessed early clinical evidence of antitumor activity of this combination in patients with HER2+ solid tumors.

## Methods

### Study design and treatment plan

This phase 1, multicenter, open-label, nonrandomized, dose-defining study was conducted in accordance with the Declaration of Helsinki and the International Conference on Harmonisation Good Clinical Practice Guidelines, and was approved by relevant regulatory and independent ethics committees including Memorial Sloan-Kettering Cancer Center’s Institutional Review Board, Mofftt Cancer Center’s Quorum Review Institutional Review Board, and The National Research Ethics Service, The Royal Marsden Research Ethics Committee. Patients provided written consent prior to enrolling in the trial. The primary objective of the study was to determine the safety and tolerability, define the DLTs and MTD, and determine the recommended phase 2 dose of MK-2206 in combination with trastuzumab. Dose finding was based on toxicity probability intervals [14]. In brief, three patients were first dosed at each level and advanced according to the toxicity probability interval; up to another 10 patients (total of 13 patients at a dose level) could be assigned to one dose, in which case up to four DLT events in the dose level of 13 patients would be considered tolerable. Secondary objectives of the trial were to explore the antitumor activity and pharmacokinetics of MK-2206 in combination with trastuzumab in patients with advanced HER2+ solid tumors. Correlation of antitumor activity with PI3K pathway activation events (that is, circulating tumor DNA and mutations) was an exploratory objective of this trial.

Trastuzumab 8 mg/kg was administered as a standard intravenous infusion (loading dose) on day 1 followed by 6 mg/kg every 3 weeks. Oral MK-2206 was given either as a 45 mg or 60 mg dose QOD in two cohorts, or as a 135 mg and 200 mg dose QW in two cohorts; patients were enrolled in parallel in the two MK-2206 dosing schedules. Treatment continued until disease progression, development of unacceptable toxicity, or patient withdrawal of consent. Dose modification of oral MK-2206 was permitted for patients who experienced grade 2 or higher drug-related toxicities (that were not clinically manageable) after the first cycle of treatment. No dose modifications were planned for trastuzumab during the course of the study, unless a patient experienced a toxicity not specified in the protocol. Patients were evaluated every 3 months by computed tomography or magnetic resonance imaging scans. Overall tumor response and progression were evaluated according to the Response Evaluation Criteria in Solid Tumors guidelines (RECIST v1.0).

### Patient eligibility

Patients 18 years of age or older with Eastern Cooperative Oncology Group performance status 0 to 1 and adequate hematologic, kidney, and liver function, and with histologically or cytologically confirmed locally advanced or metastatic HER2+ solid tumors, were eligible for the trial. Patients with significant cardiac disease or known active central nervous system metastases and/or carcinomatous meningitis were not eligible – unless they had completed radiation or were clinically stable for 1 month prior to entry without evidence of new or enlarging central nervous system metastasis, and were no longer taking steroids for brain edema. Patients who were receiving trastuzumab and/or lapatinib prior to screening had to be off both drugs for 1 week prior to enrollment if trastuzumab was administered at 2 mg/kg per week, or for 3 weeks if trastuzumab was administered at 6 mg/kg per week; other chemotherapeutic or experimental agents were not allowed within 30 days of entering the trial. Since MK-2206 is metabolized by cytochrome p450 3A4, patients using potent cytochrome p450 3A4 inhibitors or inducers had to be off the medication for at least 14 days before the first dose of the study medications. Pregnant or lactating women were not eligible for enrollment.

### Safety, tolerability, and toxicity assessment

Patients were evaluated at baseline and throughout the course of the study using physical examinations, vital signs, ophthalmologic examinations, 12-lead electrocardiogram cardiac monitoring, laboratory tests, and Eastern Cooperative Oncology Group performance status. Patients were also monitored carefully for the development of adverse events (AEs) during treatment with MK-2206 in combination with trastuzumab; AEs were graded according to the National Cancer Institute Common Terminology Criteria for Adverse Events version 3.0. Hematological and nonhematological DLTs occurring within the first 21 days of cycle 1 were used to determine the MTD of MK-2206 in combination with trastuzumab. Hematological DLTs were defined as grade 4 neutropenia lasting 5 days or more, grade 3 or 4 neutropenia with fever >38.5°C and/or infection requiring antibiotic or antifungal treatment, and grade 4 thrombocytopenia (≤25.0 × 10^9^/l). Nonhematological DLTs included any grade 3 or higher toxicity, with the specific exception of grade 3 nausea, vomiting, diarrhea, or dehydration with inadequate treatment lasting <48 hours; asthenia; inadequately treated hypersensitivity reactions; grade 3 elevated transaminases lasting <1 week; and isolated nonfasting grade 3 glucose without adequate supportive care measures. Additional DLTs included any drug-related AE, regardless of National Cancer Institute Common Terminology Criteria for Adverse Events grade, leading to a dose modification of MK-2206 in the first cycle; unresolved grade 2 or higher drug-related AEs requiring drug interruption for 8 days or more in the first cycle; and unresolved drug-related AEs requiring drug interruption for a total of 15 days or more in the first cycle.

### Pharmacokinetic and nucleic acid analysis

Sampling for pharmacokinetic determinations was conducted in all patients from each dose level during the first and second cycles of therapy. Plasma samples were collected to determine concentrations of MK-2206 on day 1 predose and at 2, 4, 6, 10, 24 (predose day 2), and 48 hours (predose day 3) after the first dose of study medication for cycle 1 and cycle 2. On days 7 and 15 of cycle 1, samples were collected immediately prior to the administration of MK-2206. Plasma concentration of MK-2206 was used to determine pharmacokinetic parameters, including the peak plasma concentration (C_max_), time to maximum concentration, minimum plasma concentration, and area under the concentration–time curve (AUC), as described previously by Yap and colleagues [13].

We requested that all patients enrolled in this study submit formalin-fixed, paraffin-embedded tumor tissue for analysis of loss of *PTEN* and mutations in *PIK3CA* and related genes. A separate fresh whole blood sample was collected at baseline to isolate circulating tumor nucleic acids in order to detect mutations in *PIK3CA*, specifically codons encoding amino acids E542, E545, and H1047.

### Statistical analyses

Since the primary objective of the trial was to determine the safety and tolerability of MK-2206 in combination with trastuzumab, the trial sample size depended primarily on clinical rather than statistical considerations. Specifically, the final number of subjects enrolled in the study was dependent on empirical safety (DLT) observations. All patients who received at least one dose of study treatment were assessed for safety data, and descriptive tables summarizing the number and percentage of patients who experienced AEs were generated. No efficacy target was predefined since antitumor activity was a secondary objective of the trial. The response rate and 95% confidence intervals were determined for response to treatment data, and summary statistics were generated for pharmacokinetic data.

## Results

### Patient characteristics

**Figure 1:**
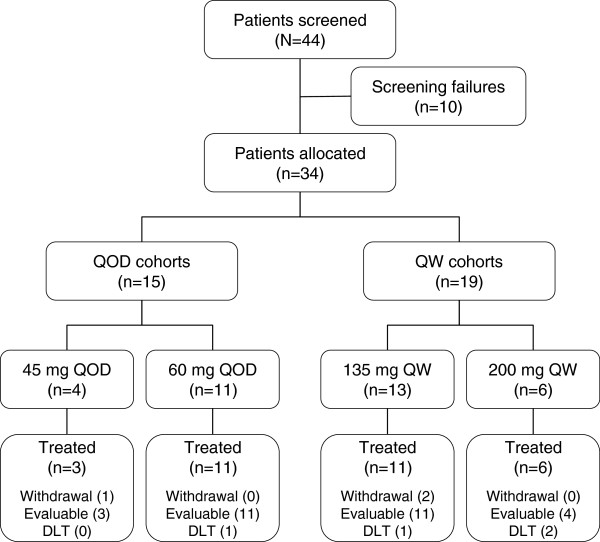
**Patient flowchart.** DLT, dose-limiting toxicity; QOD, every other day; QW, every week.

From September 2009 to February 2011, 44 patients were screened at three participating sites; 34 were enrolled in the trial and 31 patients received study medications. Three patients (one patient allocated to the QOD cohort and two patients allocated to the QW cohort) withdrew consent prior to receiving the first dose of treatment. Among the 31 patients treated, three patients were in the 45 mg QOD cohort, 11 patients were in the 60 mg QOD cohort, 11 patients were in the 135 mg QW cohort, and six patients were in the 200 mg QW cohort (Figure 1). There were 27 patients with breast cancer and four patients with gastric cancers, and the majority of patients (93.5%) had received at least three prior lines of therapy. Table 1 summarizes the demographics and baseline characteristics of the 31 patients who were enrolled in the trial and received treatment.

**Table 1:** Demographics and baseline characteristics for patients treated in the trial

**Baseline characteristic**	**MK-2206 dosing cohort**				
	**45 mg QOD**	**60 mg QOD**	**135 mg QW**	**200 mg QW**	**All cohorts**
	**(*n*** **= 3)**	**(*n*** **= 11)**	**(*n*** **= 11)**	**(*n*** **= 6)**	**(*N*** **= 31)**
Gender, *n* (%)					
Male	0 (0.0)	1 (9.1)	0 (0.0)	2 (33.3)	3 (9.7)
Female	3 (100.0)	10 (90.9)	11 (100.0)	4 (66.7)	28 (90.3)
Age (years)					
Median	44	55.5	49	48.5	51.5
Range	36 to 52	44 to 67	37 to 61	38 to 59	36 to 67
Cancer type,* n *(%)					
Breast	3 (100.0)	10 (90.9)	11 (84.6)	3 (50.0)	27 (87.1)
Gastric	0 (0.0)	1 (9.1)	0 (0.0)	3 (50.0)	4 (12.9)
Cancer stage,* n *(%)					
Intravenous	3 (100.0)	11 (100.0)	11 (100.0)	6 (100.0)	31 (100.0)
ECOG status,* n *(%)					
0	3 (75.0)	7 (63.6)	10 (91.0)	4 (66.7)	24 (77.4)
1	0 (0.0)	4 (36.4)	1 (9.0)	2 (33.3)	7 (22.6)
Number of prior therapies,* n *(%)					
1 or 2	0 (0.0)	0 (0.0)	0 (0.0)	2 (33.3)	2 (6.5)
≥3	3 (100.0)	11 (100.0)	11 (100.0)	4 (66.7)	29 (93.5)

ECOG, Eastern Cooperative Oncology Group; QOD, every other day; QW, every week.

### Treatment tolerability

The combination of trastuzumab and MK-2206 was generally well tolerated. Based on prior experience with monotherapy, the QOD dosing schedule was tested in two cohorts of 45 mg and 60 mg QOD, and the QW cohorts were tested at 135 mg and 200 mg. There were no DLTs in the 45 mg QOD cohort, but among the 11 patients treated with 60 mg QOD one patient developed a DLT (grade 2 skin rash requiring dose modification). Among the 11 patients in the 135 mg QW cohort, one patient experienced two DLTs of grade 1 and grade 2 skin rash requiring dose modification. Among the six patients in the 200 mg QW cohort, two patients were not evaluable due to rapid progression and were taken off the study before the end of first cycle; two of the four evaluable patients developed DLTs (one with grade 2 maculopapular skin rash, and one with grade 3 skin rash and grade 3 hypersensitivity). The 200 mg QW cohort was therefore judged not to be tolerable in this population of heavily pretreated breast and gastroesophageal cancer patients. Due to the early termination of the trial when the sponsor withdrew support, we were not able to determine a true MTD for MK-2206. However, the 60 mg QOD and 135 mg QW doses may represent the MTD in combination with the standard dose of trastuzumab administered (although the true MTD may be higher).

Among the patients treated, the most common treatment-emergent AEs occurring in at least 40% of patients in any treatment group included fatigue, hyperglycemia, rash, nausea, increase in liver enzymes, and decreased appetite (Table 2). There was one reported death during the course of the trial (malignant neoplasm), which was considered unrelated to treatment with MK-2206 in combination with trastuzumab. No clear patterns or apparent differences in adverse experiences were observed between the 60 mg QOD and 135 mg QW cohorts.

**Table 2:** **Treatment-emergent adverse events occurring in ≥30% of patients treated with MK-2206 plus trastuzumab (*n*** **= 31)**

**Adverse event**	**All grades (%)**	**Grade 3 (%)**	**Grade 4 (%)**
Fatigue	71.0	0	0
Hyperglycemia	51.8	6.5	0
Rash	58.1	16.7	0
Nausea	48.4	0	0
Alkaline phosphatase increase	45.2	6.5	0
Aspartate aminotransferase increase	45.2	9.7	0
Decreased appetite	41.9	0	0
Diarrhea	35.5	0	0
Alanine aminotransferase increase	32.3	3.2	3.2
Decreased hemoglobin	32.3	3.2	0
Dyspepsia	32.3	0	0
Hypoalbuminenia	32.3	0	0
Vomiting	32.3	0	0

### Clinical response

**Figure 2:**
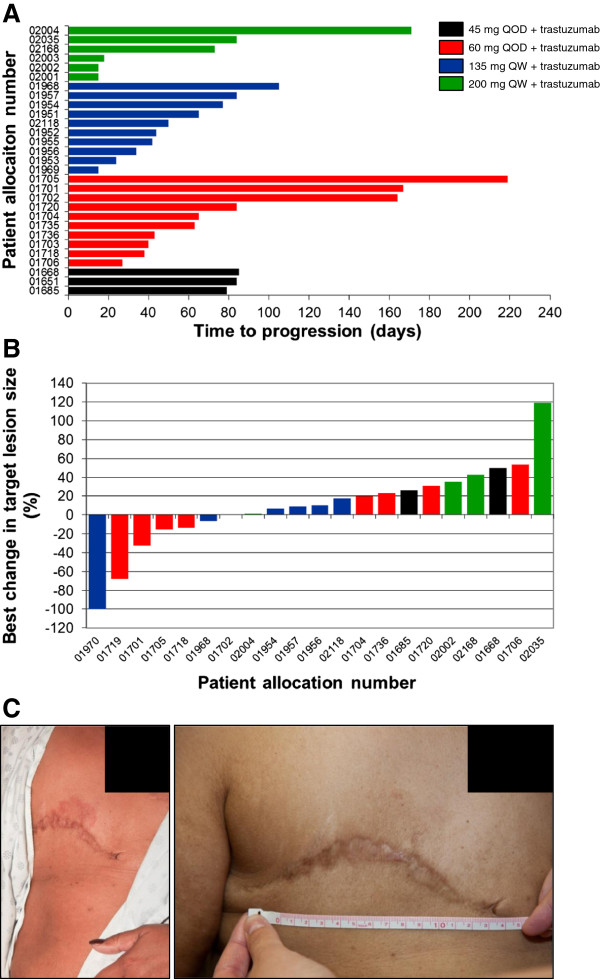
**Activity of MK-2206 following treatment with the combination of MK-2206 and trastuzumab in patients with human epidermal growth factor receptor 2-positive breast cancer or gastric cancer. (A)** Time to progression for all patients enrolled who progressed during the trial and **(B)** best change in the size of target lesions from baseline, following treatment with the combination of MK-2206 and trastuzumab in patients with human epidermal growth factor receptor 2-positive breast cancer or gastric cancer. One patient achieved complete response and one patient had partial response; both patients withdrew from the study due to adverse events and are not included in **(A)**. In **(B)**, Patient 01719 had a 41% decrease in tumor size while on trial and achieved an overall 68% decrease in the size of target lesions while continuing to receive treatment after discontinuation of the study. **(C)** One patient with breast cancer who achieved complete response had an area of erythematous skin lesion on the operative site at baseline (left panel) that completely resolved after receiving two cycles of treatment at the 60 mg every other day (QOD) MK-2206 dose level (right panel). QW, every week.

Among the evaluable patients who were treated with study medications for at least one cycle, one patient achieved complete response (CR), one patient had partial response (PR), and five patients had stable disease (SD) for 4 months or longer (one SD patient treated for over 5 months had unconfirmed PR by the investigators). Based on data collected by the time of discontinuation of the study (database lock), the clinical benefit response rate (CR, PR, and SD ≥4 months) was determined to be 24%, and the median time to progression was 72 days (range 13 to 173 days). One patient, who continued treatment after database lock, received 18 cycles of therapy (for a total of 388 days) before discontinuing due to skin rash. Details of patients who responded to treatment are presented in Table 3; all patients who achieved CR and PR had breast cancer, and a single male patient with gastric cancer achieved SD as a best response. The patient with gastric cancer had not received trastuzumab in the past, while all the other patients with breast cancer had progressed on trastuzumab with an interval from the last dose of trastuzumab of 0 to 68 days. The one patient with CR had metastatic breast cancer with progressive chest wall lesions while on trastuzumab; during the course of our study, the metastatic skin lesions completely resolved after two cycles of treatment. Unfortunately, this patient elected to stop receiving treatment due to a flare-up of ulcerative colitis (possibly related to treatment) during cycle 6 of treatment. The time to progression for all enrolled patients and the best target lesion response are depicted in Figure 2.

**Table 3:** Best tumor response in HER2+ breast and gastric patients following treatment with MK-2206 and trastuzumab

**Patient**	**Age (years)**	**Sex**	**Diagnosis**	**MK-2206 dose**	**Prior lines of therapy**	**Trastuzumab-free interval (days)**	**Reason for discontinuing prior trastuzumab**	**Time to progressive disease/withdrawal (days)**	**Best response**
A	40 to 49	Female	Breast cancer	60 mg QOD	5	41	Progressive disease	167	SD (unconfirmed PR)
B	50 to 59	Female	Breast cancer	60 mg QOD	4	21	Progressive disease	164	SD
C	50 to 59	Male	Gastric cancer	200 mg QW	2	0	Trastuzumab not given	171	SD
D	50 to 59	Female	Breast cancer	60 mg QOD	8	48	Unknown	219	SD
E	40 to 49	Female	Breast cancer	135 mg QW	5	68	Completed therapy	105	SD
F	50 to 59	Female	Breast cancer	60 mg QOD	3	0	Progressive disease	388 (withdraw)^a^	PR
7	50 to 59	Female	Breast cancer	135 mg QW	5	28	Progressive disease	106 (withdraw)	CR

CR, complete response; HER2, human epidermal growth factor receptor 2; PR, partial response; QOD, every other day; QW, every week; SD, stable disease. ^a^Patient was on trial for 173 days and then continued receiving treatment after database lock for a total of 388 days before withdrawing due to skin rash.

### Pharmacokinetics

**Figure 3:**
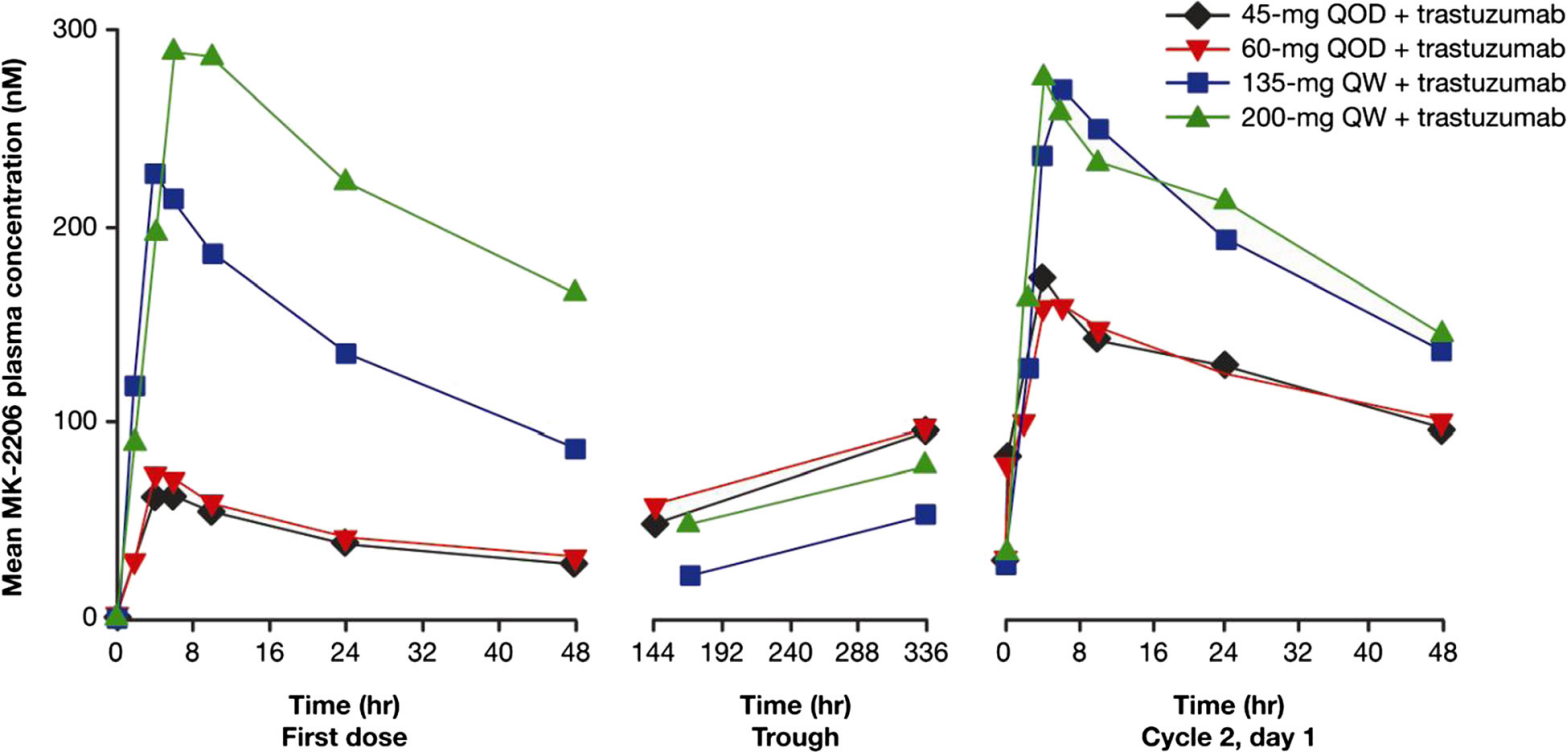
**Mean MK-2206 plasma concentration profiles following administration of MK-2206 in combination with trastuzumab.** Linear scale of plasma concentration following administration of MK-2206, in every other day (QOD) and every week (QW) schedules, in combination with trastuzumab.

Mean plasma concentrations of MK-2206 administered as 45 mg or 60 mg QOD doses and as 135 mg or 200 mg QW doses with a standard dose regimen of trastuzumab are shown in Figure 3. MK-2206 was absorbed, with median time to maximum concentration ranging from 4 to 6 hours after co-administration of 45 mg or 60 mg QOD doses of MK-2206 with trastuzumab, and 4 to 8 hours after 135 mg and 200 mg QW doses of MK-2206 with trastuzumab. Interindividual variability of plasma concentrations were moderate to high; a small number of patients received a 45 mg MK-2206 QOD dose (*n* = 3); only two patients completed dosing of 200 mg MK-2206 QW and pharmacokinetic sampling in cycle 1 as scheduled. The mean accumulation ratio of C_max_ and AUC_0 to 48 hours_ after multiple doses over 21 to 24 days, expressed as the geometric mean ratio last dose/first dose, ranged from 2.34 to 2.76 for the 45 mg and 60 mg QOD doses. The effective half-life determined from C_max_ and AUC accumulations ratio ranged from 60 to 96 hours, consistent with the mean terminal half-life of 63 to 89 hours for MK-2206 alone, and suggests that elimination of MK-2206 was not altered after co-administration with trastuzumab. The geometric mean ratios of C_max_ after multiple dosing for 21 to 22 days (last dose/first dose) for patients on the 135 mg and 200 mg MK-2206 QW dosing schedule were 1.26 and 1.30, respectively. The effective half-life based on accumulation ratio of C_max_ and AUC was 74 to 79 hours. Similar geometric mean ratios were obtained for AUC_0 to 48 hours_ after QW dosing with trastuzumab. The pharmacokinetic results were consistent with data from a prior monotherapy study of MK-2206, suggesting that trastuzumab did not appreciably alter the pharmacokinetics of MK-2206. In addition, the trough levels of all patients (11/11) receiving 45 mg or 60 mg QOD doses of MK-2206 with trastuzumab was at or above the clinical monotherapy efficacy trough target of 56.8 nM. Similarly, 10 out of 11 (~92%) patients receiving 135 mg or 200 mg QW doses of MK-2206 also achieved the 48-hour target of at least 56.8 nM.

### Circulating nucleic acid

All patients enrolled in the study had a baseline blood sample collection for analysis of circulating nucleic acid for mutations in *PIK3CA*. Only three of the 37 patients enrolled were found to have *PIK3CA* gene mutations: two patients with breast cancer who went on to receive treatment had an H1047L mutation in exon 9 and an E545K mutation, and the third patient had a less predominant M1043I mutation but withdrew prior to documentation of progression of disease.

## Discussion

Trastuzumab is effective therapy for HER2+ breast cancers and gastric cancers. However, relative resistance to trastuzumab is common via multiple mechanisms [5-8]. Through unbiased RNA interference screening analyses, activation of the PI3K pathway has been implicated as a major mediator of trastuzumab resistance [7]. Based on these data and preclinical findings that HER2 signaling is highly dependent on PI3K/AKT signaling [10], we hypothesized that tumors could have compensatory activation of this pathway, thereby avoiding the impact of HER2 inhibitors. To begin clinical exploration of combined HER2 and AKT signaling blockade, we evaluated treatment with trastuzumab and the allosteric AKT inhibitor MK-2206 in this phase 1 study. Previously, monotherapy with MK-2206 given either QOD or QW was tolerable, leading us to examine both dosing schedules combined with trastuzumab [13,15]. The majority of patients enrolled in the study had exposure to trastuzumab and had progressed on treatment. Our study demonstrated that the combination of trastuzumab and MK-2206 was as tolerable as the same dosing schedule using MK-2206 monotherapy, with no evidence of enhanced toxicities with combined therapy. A true MTD for MK-2206 in combination with trastuzumab was not established, but the 60 mg QOD and 135 mg QW doses are reasonable doses for future evaluation in phase 2 trials. The pharmacokinetic profile of MK-2206 in this study was similar to that observed when MK-2206 was administered as monotherapy. In addition, the DLT of the combination therapy was skin rash, which was the same as the DLT of MK-2206 given as monotherapy. Other observed AEs were also consistent with those of MK-2206 single-agent therapy.

The combination of MK-2206 and trastuzumab also demonstrated preliminary evidence of therapeutic efficacy in patients with HER2+ breast cancer or gastroesophageal cancer, with a clinical benefit response rate of approximately 24% and a median time to progression of 72 days. One patient with metastatic breast cancer, whose disease progressed on the right chest wall around the previous mastectomy scar while on maintenance therapy with trastuzumab, achieved CR following combination therapy with MK-2206. Her erythematous chest wall skin lesion showed a dramatic improvement after receiving two cycles of study treatment and by 6 months the skin lesion had completely resolved. There was one additional patient with breast cancer treated for over a year experiencing a total reduction in tumor size of 68% who was confirmed as having PR. Five more patients had SD for more than 4 months. These preliminary efficacy results suggest that the combination of MK-2206 with trastuzumab may offer patients an effective salvage regimen following progression on trastuzumab, or may prevent or delay clinical resistance if used earlier in the disease.

The efficacy observed in this phase 1 study supports the hypothesis that a mechanism of resistance to trastuzumab may be mediated by activation of the PI3K/AKT pathway *in vivo*. The mechanisms through which the PI3K/AKT pathway may be activated in trastuzumab-refractory HER2+ tumors is currently unknown. Leading candidates include activating mutations of the *PIK3CA* gene or deletion or mutations in *PTEN*, an inhibitor of the PI3K/AKT pathway. We collected circulating nucleic acid to explore this possibility, based on reports that correlated findings in circulating nucleic acid with DNA from tumor specimens [16]. Only three patients were found to have mutations in the *PIK3CA* gene in circulating DNA and none had notably long SD or response to treatment. No *PIK3CA* mutation was detected in the circulating nucleic acid samples from patients who responded to treatment. Studies have estimated that between 13 and 31% of HER2+ breast cancers harbor mutations in *PIK3CA*[17]. Results of *PIK3CA* mutation status from circulating DNA in this study (two of 27 HER2+ breast cancer patients who received treatment) are at the lower limit of these estimations. One of the limitations of this analysis is that our *PIK3CA* mutation assessment was restricted to circulating DNA analysis. Tumor biopsies for biomarker analysis prior to treatment were not mandated and intratumor heterogeneity in *PIK3CA* mutation status or limitations of detection inherent to circulating DNA mutational analysis may be responsible for the lower than expected *PIK3CA* mutational frequency observed. The possibility therefore remains that tumor samples at primary or metastatic sites might demonstrate mutations that do not appear in circulating nucleic acid.

Despite these caveats, our analysis of the circulating DNA *PIK3CA* somatic mutation status does not support the hypothesis that tumors with *PIK3CA* mutations have improved responsiveness to MK-2206. Conceivably, other molecular aberrations such as p95HER2, *PTEN* loss of function events or alternative signaling cascades mediated by HER3, and insulin growth factor-1 receptors or epidermal growth factor receptors that were not assessed in our study, may also be predicted to render tumors resistant to trastuzumab but sensitive to combined AKT inhibition. Therefore, in an attempt to predict preferential benefit from combined AKT inhibitor/trastuzumab therapy, exploratory biomarker analyses may need to consider the polygenic nature of trastuzumab resistance and assess multiple aberrations in the HER2 signaling pathway in each tumor. This finding is consistent with the recent report of the combination of trastuzumab and everolimus, a mammalian target of rapamycin inhibitor [18]. In that study, tumors demonstrating loss of *PTEN* were associated with poorer overall survival, although loss of *PTEN* and/or *PI3KCA* mutations did not seem to affect progression-free survival, compared with those without genetic alterations. Additional studies are needed to generate more data to fully determine the potential role of circulating DNA mutations as predictors of drug sensitivity in this population.

Numerous agents specifically targeting dysregulated molecular pathways, believed to be key tumorigenic drivers, have recently been approved or are being evaluated as potential treatment options in patients with breast cancer or other tumor types [19]. Combined antibody therapy, using both trastuzumab and chemotherapy with or without pertuzumab, was recently shown to be effective [20]. However, nonchemotherapeutic approaches are attractive because they promise reduced toxicity. For example, a phase 3 trial evaluated the combination of trastuzumab and the small molecule, reversible inhibitor of epidermal growth factor receptor and HER2, lapatinib, in HER2+ metastatic breast cancer patients refractory to trastuzumab administered in the absence of chemotherapy [21]. Results from this trial, which enrolled 296 patients, demonstrated improvements in overall survival, progression-free survival and clinical benefit response in the combination arm compared with treatment with lapatinib alone. However, the difference in median progression-free survival – specifically between the two treatment arms – was only 4 weeks (12.0 weeks in the combination group vs. 8.1 weeks in the lapatinib monotherapy group), and the majority of patients did not achieve a dramatic improvement in tumor response rate or survival, suggesting that the combined blockade of HER2 signaling is active even without chemotherapy, but may not be sufficient to overcome downstream PI3K/AKT pathways responsible for resistance to trastuzumab.

Based on results from our phase 1 study, we believe that additional translational studies of MK-2206 with trastuzumab and possibly other agents including pan-HER kinase inhibitors (for example, lapatinib or neratinib) or broad cytotoxic agents (for example, paclitaxel) are warranted. Treatment with MK-2206 has been shown to upregulate HER3 via feedback mechanisms limiting antitumor effects, which could be rescued by the addition of lapatinib [11]. Early phase clinical trials are already underway investigating the combination of MK-2206 and lapatinib in patients with advanced or metastatic solid tumors or breast cancer.

## Conclusions

Our results show evidence of antitumor activity in patients with HER2+ breast cancer and gastroesophageal cancer following treatment with standard doses of trastuzumab and MK-2206, and the combination was generally well tolerated. Trastuzumab did not affect the pharmacokinetic profile of MK-2206, suggesting that this AKT inhibitor can be safely combined with trastuzumab. Our results support further investigations with MK-2206 in combination with HER2 inhibitors or cytotoxic agents for patients with treatment-refractory HER2+ tumors.

## Abbreviations

AE: adverse event; AUC: area under the concentration–time curve; Cmax: peak plasma concentration; CR: complete response; DLT: dose-limiting toxicity; HER2: human epidermal growth factor receptor 2; MTD: maximum tolerable dose; PI3K: phosphatidylinositol-3 kinase; PR: partial response; QOD: every other day; QW: once weekly; SD: stable disease.

## Competing interests

CH received a grant (paid directly to his institution) for the conduct of the study. CS has served as an advisor and received consulting fee or honoraria from Merck. YYJ has served as a consultant for Genentech/Roche. R Lee was an employee of and owned stock in Merck at the time the study was conducted. SC has served as a consultant for Merck. JS is an employee of Merck. JK, KS, ET, and LY are employees of and own stock in Merck. HSH has received research grants from the American Cancer Society and Merck. SS, R Leh, NH, DG, and CM have nothing to disclose related to this work. Medical writing and editorial assistance was provided by Kakuri M Omari, PhD, of Integrus Scientific, a division of Medicus International New York (NY, USA) entirely before 1 June 2013. (They did not provide any further editorial assistance including with subsequent revisions). This assistance was funded by Merck Sharp & Dohme Corp., a subsidiary of Merck & Co., Inc. (Whitehouse Station, NJ, USA). The authors were fully responsible for all content and editorial decisions and received no financial support or other compensation related to the development of the manuscript.

## Authors’ contributions

CH designed and planned the study, collected and assembled data, supervised analyses, interpreted the results, wrote sections of the initial draft, provided substantive suggestions for revision and critically reviewed later drafts, provided study materials and patients, obtained funding, and provided administrative, technical, and logistical support. CS collected and assembled data, interpreted the results, wrote sections of the initial draft, and provided substantive suggestions for revision and critically reviewed later drafts. YYJ collected and assembled data, interpreted the results, provided substantive suggestions for revision and critically reviewed later drafts, provided study materials and patients, and provided administrative, technical, or logistical support. RLee collected and assembled data, and critically reviewed later drafts. SS collected and assembled data, critically reviewed later drafts, and provided study materials. RLeh collected or assembled data, critically reviewed later drafts, and provided administrative, technical, and logistical support. SC conceived, designed, and planned the study, interpreted the results, and provided substantive suggestions for revision. NH collected or assembled data and critically reviewed later drafts. DG interpreted the results, provided substantive suggestions for revision and critically reviewed later drafts, and provided study materials and patients. JK planned the study, assembled data, and critically reviewed later drafts. JS assembled data, performed analyses, interpreted the results, and provided substantive suggestions for revision. KS interpreted the results and critically reviewed later drafts. ET interpreted the results, and wrote sections of the initial draft. DMS interpreted the results and critically reviewed later drafts. CM assembled data, critically reviewed later drafts, and provided study materials and patients. LY collected data, critically reviewed later drafts, provided study materials and patients, and obtained funding. HSH collected data, interpreted the results, provided substantive suggestions for revision and critically reviewed later drafts, and provided study materials and patients. All authors read and approved the final manuscript.
